# The Application of Microfluidic Chips in Primary Urological Cancer: Recent Advances and Future Perspectives

**DOI:** 10.1002/smmd.70010

**Published:** 2025-05-19

**Authors:** Jiafu Liu, Xiao Zhi, Xiaolan Fang, Wenyao Li, Weixin Zhao, Meng Liu, Enping Lai, Wenzhuo Fang, Juan Wang, Yu Zheng, Jiang Zou, Qiang Fu, Wenguo Cui, Kaile Zhang

**Affiliations:** ^1^ Department of Urology Affiliated Sixth People's Hospital Shanghai Jiao Tong University School of Medicine Shanghai China; ^2^ School of Materials Science and Engineering Shanghai University of Engineering Science Shanghai China; ^3^ Institute for Personalized Medicine School of Biomedical Engineering Shanghai Jiao Tong University Shanghai China; ^4^ National Engineering Research Center for Nanotechnology Shanghai China; ^5^ Department of Pathology Henry Ford Hospital Detroit Michigan USA; ^6^ Wake Forest Institute for Regenerative Medicine Winston‐Salem North Carolina USA; ^7^ Guangxi Key Laboratory of Green Processing of Sugar Resources College of Biological and Chemical Engineering Guangxi University of Science and Technology Liuzhou China; ^8^ Department of Orthopaedics Shanghai Key Laboratory for Prevention and Treatment of Bone and Joint Diseases Shanghai Institute of Traumatology and Orthopaedics Ruijin Hospital Shanghai Jiao Tong University School of Medicine Shanghai China; ^9^ School of Mechanical Engineering Shanghai Jiao Tong University Shanghai China; ^10^ State Key Laboratory of Mechanical System and Vibration Shanghai Jiao Tong University Shanghai China

**Keywords:** in vitro model, microfluidic technology, organ‐on‐a‐chip, urological cancer

## Abstract

The research of primary urological cancers, including bladder cancer (BCa), prostate cancer (PCa), and renal cancer (RCa), has developed rapidly. Microfluidic technology provides a good variety of benefits compared to the heterogeneity of animal models and potential ethical issues of human study. Microfluidic technology and its application with cell culture (e.g., organ‐on‐a‐chip, OOC) are extensively used in urological cancer studies in preclinical and clinical settings. The application has provided diagnostic and therapeutic benefits for patients with urological diseases, especially by evaluating biomarkers for urinary malignancies. In this review, we go through the applications of OOC in BCa, Pca and Rca, and discuss the prospects of reducing the cost and improving the repeatability and amicability of the intelligent integration of urinary system organ chips.

## Graphical abstract

The researches of primary urological cancer include bladder cancer (BCa), prostate cancer (PCa), and renal cancer (RCa) developed rapidly but with many drawbacks. Compared to the heterogeneity of animal models and the ethical issues of human study, microfluidic technology provides various benefits. Microfluidic technology and its application with cell culture (organ‐on‐a‐chip, OOC) have been extensively applied to study urological oncology in preclinical and clinical settings. These studies have broadened the diagnostic and therapeutic approaches for urological diseases, especially for evaluating biomarkers for urinary malignancies. In the future, microfluidic and OOC are expected to extensively complement animal models and clinical research. For the prospection of urological microfluidic studies, how to reduce costs and improve intelligent integration reproducibility and bionics are wanted.

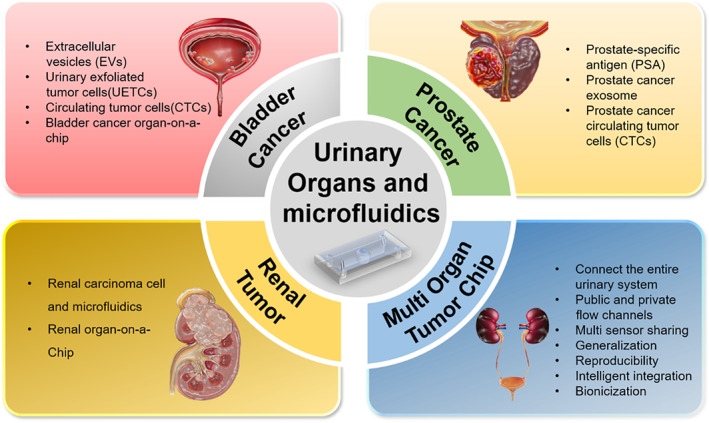

**Summary**

- The first comprehensive and systematic review of the current research studies in microfluidic and organ‐on‐a‐chip (OOC) technologies in urological cancer.
- The review focuses on three major urological types of neoplasm: bladder cancer, prostate cancer and renal cancer.

**Abbreviations**
2D: Two Dimensions
2.5D: Two and a Half Dimensions
3D: Three Dimensions
5‐FU: 5‐fluorouracil
ADT: androgen deprivation therapy
AFM: Atomic Force Microscopy
AI: artificial intelligence
anti‐EGFR: anti‐Epidermal Growth Factor Receptor
ASR: Age‐standardized incidence rate
BCa: Bladder Cancer
BCG: *Bacillus* Calmette‐Guerin
BCOC: Bladder cancer‐on‐a‐chip
BTA: Bladder Tumor Antigen
Caco‐2: Human colon adenocarcinoma cell line Caco‐2
Caki: Human Renal Cell Carcinoma Cell Lines Caki
Caki‐1: Human Renal Clear Cell Carcinoma Cell Line Caki‐1
CD55: Complement decay‐accelerating factor
CD63: Cluster of Differentiation 63
cfDNA: cell‐free DNA
CIP: Ciprofloxacin
CK: Cytokeratin
CT: Computed Tomography
CTCs: Circulating tumor cells
DNA: Deoxyribonucleic Acid
DU145: DU 145 Human Prostate Carcinoma Cell Line
EBCCs: Exfoliated BCa cells
EGFR: Epidermal growth factor receptor
ELISA: Enzyme‐Linked Immunosorbent Assay
EMT: Epithelial‐mesenchymal transition
EpCAM: Epithelial Cell Adhesion Molecule
ETCs: exfoliated tumor cells
EVs: Extracellular vesicles
FDA: Food and Drug Administration
GelMA: Gelatin Methacryloyl
GKA: gut‐kidney axis
H&E staining: Hematoxylin and Eosin staining
HepLL cells: hepatocytes
HKC‐8: Human Kidney Proximal Tubular Epithelial Cells HKC‐8
HSP90: Heat shock protein 90
HUS: hemolytic uremic syndrome
HUVEC: Human Umbilical Vein Endothelial Cell
LNCaP: Lymph Node Carcinoma of the Prostate
lncRNAs: long non‐coding RNAs
Mag‐CD63: anti‐CD63 magnetic nanoparticles
MARCKS: myristoylated alanine‐rich C‐kinase substrate
MARCKSL1: MARCKS‐related protein
mCRPC: metastatic castration‐resistant PC
miRNAs: microRNAs
MRC‐5: Medical Research Council cell strain 5
MSC: Mesenchymal Stem Cell
NEnC: Normal endothelial cells
NMP22: Nuclear Matrix Protein 22
OOC: Organ‐on‐a‐chip
PBS: Phosphate buffered saline
PC3: Prostate Cancer Cell line 3
PCa: Prostate Cancer
PDMS: Polydimethylsiloxane
PDXs: Patient‐derived xenografts
PLGA‐PEG: Poly(lactic‐co‐glycolic acid)‐Polyethylene glycol
POC: Point‐of‐care
PSA: Prostate Specific Antigen
RCa: Renal Cancer
RCC: Renal cell carcinoma
RNA: Ribonucleic Acid
RT‐qPCR: Reverse Transcription Quantitative Polymerase Chain Reaction
SDC1: Syndecan‐1
Sialyl‐Tn: Sialyl Thomsen‐Friedenreich Antigen
STEC: Shiga toxin‐producing *Escherichia coli*
streptavidin‐HRP: Streptavidin‐Horseradish Peroxidase
Stx2: Shiga toxin 2
Stxs: Shiga toxins
SU‐8: Styrene‐Urethane Acrylate
TEnCs: Tumor endothelial cells
THP‐1: Human Acute Monocytic Leukemia Cell Line THP‐1
TJP2: Tight junction protein 2
TME: Tumor Microenvironment
UETCs: Urinary exfoliated tumor cells
UV: Ultraviolet
VIM: vimentin
Wi‐Fi: Wireless Fidelity

## 1 Introduction

The primary urinary system‐related neoplasms include bladder cancer (BCa), prostate cancer (PCa), and renal cancer (RCa). It is estimated to have 573,000 new cases and 213,000 related deaths in patients with BCa every year [1]. Because of the high recurrence potential, long‐term follow‐up examinations of BCa are usually required. Compared to cystoscopy, an invasive clinical test which is considered the gold standard method [2], novel non‐invasive diagnostic procedures are highly preferred. PCa is the most commonly diagnosed cancer, and the second leading cause of cancer‐related deaths in males in the US [3]. In 2020, it was anticipated that there would be more than 1,144,000 new cases of PCa per year globally, with an age‐standardized incidence rate (ASR) of 31 per 100,000. The estimated global PCa‐related deaths exceeded 375,000 per year, with the overall death rate ASR at 7.7 per 100,000 [4]. Combined evaluation of prostate‐specific antigen (PSA) levels and prostate biopsy continues to be the standard of care for PCa diagnosis. Prostate biopsy is also an invasive test and usually has unsatisfactory efficiency and accuracy [5]. RCa is a high‐risk malignancy, with metastases found in up to 25% of patients at initial diagnosis [6]. It accounts for around 5% of new cancer cases in males and 3% in females in the US [7]. There are variable classification systems of renal cell carcinoma, which consequently result in many types and respective treatments [8]. To improve the detection sensitivity and accuracy of urinary neoplasms, continuous efforts have been made to develop new approaches such as non‐invasive cancer biomarker detection [9], rapid and low‐cost detection [10], point‐of‐care (POC) diagnosis [11], and personalized treatment [12]. However, the turnaround time is usually lengthy. Thus, there is an urgent need for the advancement and evolution of new technologies, including microfluidics and organ‐on‐a‐chip (OOC) technology.

The anatomical features of the urinary system show the complexity of multiple tubular systems intertwined, which could be represented using the pipeline architecture constructed by OOC. From the key perspective of biomarker evaluation, OOC technology shows excellent characteristics and potential [13]. With its unique microfluidic system and bionic design, OOC can simulate the urodynamic state in vivo to further explore the key role of fluid mechanics‐induced mechanical‐related signaling pathways, such as AMPK‐Sirtuin1‐YAP [14]; Hippo‐YAP/TAZ [15] signaling pathway, the role of PIEZO channel family (including PIEZO1 and PIEZO2 in mammals) in mechanical force transmission and so on [16]. Those pathways play essential roles in the pathogenesis of urological disorders. The highly simulated physiological context also provides a significant prognostic value of OOC in urinary neoplasms and the potential for biomarker screening.

**FIGURE 1:**
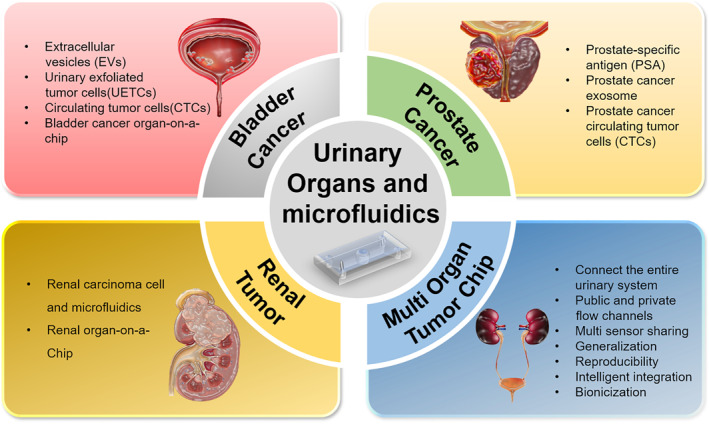
Application of microfluidics and OOCs in the detection, treatment, and prognosis of urinary system tumors.

**FIGURE 2:**
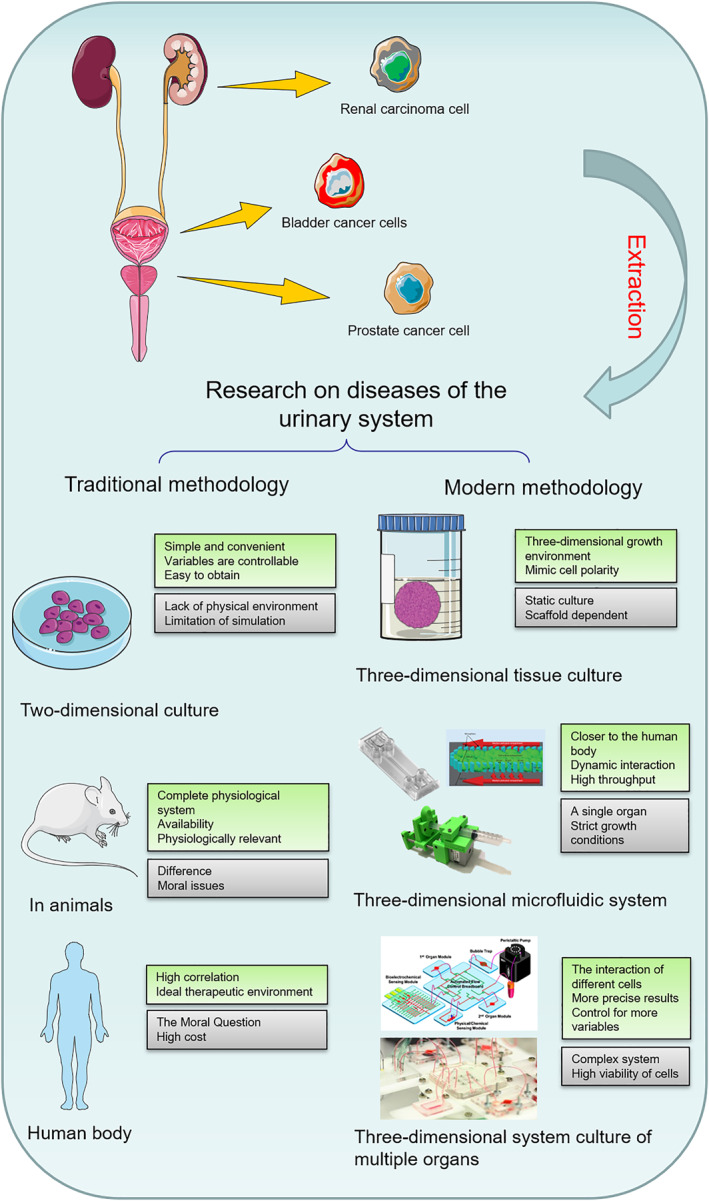
Models for diseases of the urinary system. Reproduced with permission [53]. Copyright 2017, National Academy of Sciences. Reproduced with permission [133a]. Copyright 2009, Royal Society of Chemistry. Reproduced with permission [133b]. Copyright 2018, Journal of Visualized Experiments.

The concept of application of microfluidic technology in biomedicine has been utilized in the context of associated ailments and pharmaceutical assessment on BCa and RCa. However, the application of OOC in PCa has rarely been reported, although much more microfluidic chips and OOCs are utilized in various organs outside urinary system [17]. These chips simulate the specific organ's microenvironment for functional evaluation. Some chips have mimicked the similar effect of multi‐organ collaboration (also known as multi‐organ chips) by connecting multiple single‐organ OOCs simultaneously. Currently available single‐organ OOCs include lung [17a], intestine [17b], kidney [17c], brain [17d], and heart [17e], and multi‐organ chips include liver‐intestine chips [17f] and intestine‐liver‐tumor chips [17g]. As shown in Figure 1, single‐organ chips have been developed for urinary cancers (bladder cancer, prostate cancer and renal tumor) and are expected to help with further cancer treatment. Multi‐organ chips are also in development to connect different urinary systems. Due to the diffusivity of cancer cells, multi‐organ chips could serve as an ideal in vitro model by mimicking the dynamic microenvironment, which could help with the precise treatment of cancer and other diseases of urinary organs [18]. This review highlights the development of microfluidics in the realm of the urinary system. It focuses on the research status of related urinary organ(s) on a chip, with detailed characteristics and potential challenges of microfluidic chips for urinary organs. We anticipate that this review would serve as a reference for advancing microfluidic and OOC technology, facilitating the advancement of personalized treatment strategies, and enhancing the efficacy of urinary cancer screening protocols (Figure 2).

## 2 The Composition of the Microfluidic Chip and OOC

The microfluidic chip is a microdevice that controls fluid flow at the micrometer scale. Its function largely depends on the microchannel structure inside the microchip. The OOC is a special type of microfluidic chip, also known as a microfluidic cell chip. Its main structure is generally made of biomimetic materials, and the most significant difference in composition from ordinary microfluidic chips is that it has a cell culture chamber (Table 1).

**TABLE 1:** Microfluidic and organ‐on‐chip components in the urinary system.

Components		Characteristics
Chip substrate	Polydimethylsiloxane(PDMS)	Advantages: Low cost, air permeability, transparency, good biocompatibility.
		Limitations: Untreated is hydrophobic.
		Manufacturing characteristic: Lithography, molding, etc., usually using small‐size mold for indirect manufacturing, sealing plasma surface treatment required.
	Glass	Advantages: Low cost, better transparency, good biocompatibility.
		Limitations: Inflexible, poor air permeability.
		Manufacturing characteristic: Usually, the common glass slide is used as the substrate and bonded with PDMS.
	Thermoplastic polymer	Advantages: Low cost, easy to manufacture, good rigidity.
		Limitations: Poor air permeability, poor biocompatibility.
		Manufacturing characteristic: It is easy to manufacture and can be directly 3D printed even in small sizes. Good rigidity can be used as a filter membrane or mixer component in microchips.
Cell culture system	2.5D culture	Features: Cells grow on the basement membrane, which usually requires protein surface treatment to increase cell adhesion.
	3D culture	Features: Cells are coated in a three‐dimensional culture matrix and can grow in three dimensions, which is more in line with the requirements of future organ chips.
Valves	Microvalve	Role: Microfluidic controlled switch, such as the opening and closing of the culture medium in and out.
		Features: Connecting the external catheter with the internal micro‐channel.
Micropump	Injection pump	Effect: Continuous perfusion of microfluidics in the system.
		Characteristics: Continuous perfusion of fluid, can simulate a certain shear force, the following urethral urination process or study the efficiency of the chip under different stress.
	Peristaltic pump	Effect: Intermittent perfusion of microfluidics in the most system.
		Features: Intermittent perfusion of fluid can simulate more complex body fluid microenvironment, such as the intermittent flow of urine from the upper urethra to the bladder.
Sensor	Biosensors	Features: A series of physical and chemical changes are produced by the contact with the biological body. For example, the electrical impedance sensor can classify different cancer cells by the change of potential.
	Physical sensors	Features: Real‐time monitoring of physical changes in the system, such as real‐time monitoring of cell size by optical lens, real‐time monitoring of appropriate temperature of culture system by temperature sensor.
	Chemical sensors	Features: Real‐time monitoring of chemical changes in the system, such as changes in CO_2_ gas concentration, pH value and other factors in the organs on the chip.

### 2.1 Microfluidic Chip

Microfluidic technology can regulate fluid streams in a channel in a microenvironment. When applied to an OOC, microfluidics simulates the natural flow of body fluid, mimicking the transportation of nutrients and elimination of waste liquid. With the progress of polymer materials and microsystem processing technology, polydimethylsiloxane (PDMS) microfluidic chips in biomedicine have gradually received abundant attention [19]. The PDMS chip has decent hydrophobic permeability and biocompatibility. It can precisely control various biochemical conditions through microchannels, mimicking human microenvironments with in vitro culturing of various human tissues and organoids [20].

The analytical performance of a microfluidic chip largely depends on the quality of the microchannels created inside the chip. At present, commonly used microchannel processing technologies include laser‐guided direct writing [21], photolithography [22], hot embossing [23], micro‐milling [23, 24], and 3D printing [25]. Laser‐guided direct writing uses laser ablation to directly generate three‐dimensional microchannels in glass slides without additional stacking and bonding. Photolithography and hot embossing are indirect techniques for fabricating microchannel structures, requiring the processing of molds and then the molding of microchannels [26]. Micro‐milling and 3D printing technologies can directly process micro‐channels on the surface of the workpiece or create a mold and then generate the micro‐channel structure. In photolithography, SU‐8 negative can be used as a photocurable polymer, which is solidified on the mold to produce the chip microstructure. The microstructure is then transferred to the polymer PDMS chip using the mold [27]. Positive photoresists can help sustain their sizes and patterns as the photoresist developer solvent does not permeate the areas not exposed to UV light. With negative resistance, the solvent permeates both the UV‐exposed and unexposed areas, which lead to pattern distortions. Bonding is an important link in chip fabrication. The sealing of microfluidic channels can be performed by plasma oxidation sealing [28], anodic bonding [29], ultraviolet irradiation [30], organic solvent bonding and cross‐linking agent adjustment [31]. Since PDMS relies on intermolecular forces to achieve bonding, liquid leakage is prone to occur when the pressure is high. So, the sealing between PDMS and glass chips needs to be permanent. Li et al. [32] made a microfluidic chip with PDMS‐glass, which bonded the poured PDMS to the glass with a flat and clean surface by a plasma method. They avoided the cumbersome processing of traditional glass chips as glass‐etching is not required for plasma bonding in this method. Compared with traditional etching [33] and sputtering processes [34], this method is simple, convenient, and fast with relatively low equipment requirements. It is thus considered one of the best methods for making chips concurrently [35]. Previous research found that the shear stress induced by urine flow ranges from 0.2 to 2 dyn cm^−2^ at regular flow rates, and it was reported that the bonding force can encounter the shear force in the urinary flow microenvironment [36] (Table 2).

**TABLE 2:** Comparison of different microfluidic control manufacturing technologies.

Technologies	Unique strengths	Characteristics	Application examples
Laser‐guided direct writing	– High precision – No mask required – Flexible design	– Focused laser beam to selectively deposit material (e.g., polymers or metals) – Writing directly onto substrates without the need for masks – Precision control of laser intensity and scanning speed	Progress report including laser‐guided direct writing microstructures [21a]; a 3D passive microfluidic mixer [21b, 21c]
Photolithography	– High resolution – High throughput – Established in semiconductor industry	– Coating substrate with photoresist material – Exposing the coated material to UV light through a mask – Developing the exposed material to etch patterns	Multilayer photopatterning platform for liver‐on‐a‐chip [22a]; a microfluidic device for detecting viruses using a soft lithography method [22b]
Hot embossing	– High precision – Low‐cost – Scalable for mass production	– Heating polymer material above its glass transition temperature – Applying pressure to a mold to form microstructures – Cooling and demolding the structure	Paper‐based microfluidic chips [23a]; microfluidic for vascular structure and function [23b]; microfluidic 3D cell culture platform [23c, 23d]
Micro‐milling	– High flexibility – Capable of machining complex 3D structures	– CNC‐controlled machine tools with rotating cutters – High‐speed cutting of substrates (typically metals, polymers, or ceramics) – Capable of machining intricate features at micron scale	A microfluidic device formed by micro‐milling [24a]; a liver‐on‐a‐chip platform using micro‐milling technology [24b]
3D printing	– Design freedom – Rapid prototyping – Ability to produce complex geometries	– Indirect printing: 3D printing of templates or sacrificial supports for subsequent forming of runners/complex structures – Direct printing: 3D printing fused deposition/photocuring deposition direct forming microchannel structure	3D printing for highly miniaturized and integrated microfluidics [25a]; the 3D printed microfluidic device is used for 'microfluidic biopsy' [25b, 25c]

### 2.2 From Microfluidic to OOC

OOC is a system with engineered or original tissues grown in in vitro microfluidic equipment. The microfluidic chips are designed with optimized biochemical conditions which is similar to human physiology, mainly to regulate cell microenvironments and major tissue‐specific functions on a real‐time basis [37]. It applies microfluidic chip technology in histology and is a potential substitute for future human in vitro organ models [38]. Structurally, an OOC consists of four major functional parts: an organ tissue culture chamber, a microfluidic chip, components for stimulation or drug delivery, and biosensors [39]. In addition to the advantages of tissue function and physiological structure, current OOCs could also enable the manipulation of fluids and the dynamic capture of parameter changes on the micrometer scale, further improving the simulation degree of biological models and the sensitivity to delicate experimental parameter changes.

### 2.3 The Special Cell Culture Style of OOC

Compared to conventional 2D cell culture, 2.5D and 3D cultures are most prevalent in OOC technology. They are both in a dynamic environment and usually have a variable fluid environment, while 2.5D culture mainly culture cells on the basement membrane [40]. Due to technical support and cost limitations, 2.5D culture is still primarily used in OOCs [41].

2D culture is a single layer of cells, and the 3D culture is generally an ellipsoid‐shaped cell mass. The microfluidic model of monolayer cells based on 2D culture is simplified to evaluate one or two layers of cells in a fluid environment [42]. The 3D ellipsoid in the microfluidic is more similar to the natural tissue and can better reflect the real tumor microenvironment [43]. One specific kind of OOCs provides a double‐layered membrane for two kinds of cells growing in a 2.5D environment, which allows convenient observation of the cells' connection and interaction in the pulmonary alveoli and the blood vessels [17a, 44]. When the organoids with different cells were connected in parallel or series flow channels, those organoids refer to 3D structures grown from stem cells with induced organ‐level functions, usually in a more static microenvironment, and are slightly different from the organ‐specific cells used in most of the OOC. Overall, OOCs are powerful tools for studying functional units of human organs in microfabricated cell culture devices [45].

### 2.4 Micropumps of OOC

The micropump powers the flow of culture media in the microenvironment, and fluid control is mainly driven by micropumps at the cellular level [46]. The micropumps can be classified into two types: mechanical and non‐mechanical. Mechanical micropumps include pneumatic micropumps, electromagnetic micropumps, centrifugal micropumps [47], and piezoelectric micropumps. Non‐mechanical micropumps include gravity‐driven pumps and electroosmotic‐driven pumps [48]. Currently, most simple fluid control systems use injection pumps [49] and peristaltic pumps [50] for sample transport and expulsion. For instance, the pressure of urine flow is one of the mechanical characteristics of the microenvironment of human urethra. The urine flow during the emptying process can be compared to an injection pump operating under appropriate pressure. When urine is formed and pushed from the upper urinary tract to the bladder, it could be seen as a dynamic peristaltic pump operating at the appropriate pressure. These pumps are also used to power the movement of nutrient cells needed in the microenvironment to accomplish dynamic cell culture. Dynamic liquid perfusion is another distinctive feature of microfluidic culture compared to traditional 2D culture.

### 2.5 Biosensors in OOC

At present, the activity of cells in the chip is mainly monitored using fluorescent biomarkers, whose signals could be real‐time captured for organoids and subsequent experimental operations [51]. With biosensors, the overall growth state of cells can be continuously observed. The electrical impedance sensor detects the status change of the biological element through the change of electric potential without additional labeling. An OOC developed by Osaki et al. simulated the interaction of neuromuscular junctions in 3D, and was able to record axonal growth, neuromuscular junction formation, real‐time optogenetic regulation of muscle contraction, and motor neuron activity [52]. The results strongly suggest that various neural pathological behaviors associated with muscle disease can be simulated in OOCs. In addition to the detection of potential signals, Zhang et al. developed a highly integrated modular OOC platform using the characteristics of antigen‐antibody binding. The platform has multiple sensors built‐in, including test plates for microfluidic routing through built‐in pneumatic valves, microbial reactors for organoids, physical sensing kits for measuring microenvironmental parameters, one or more electrochemical sensing units for detecting soluble biomarkers secreted by organoids, media storage and bubble traps, and fluid flows through each module. Each module in the circuit can also be replaced individually, such as the dielectric storage, bubble trap, and electrochemical sensor chip [53]. As a part of the OOC system, the existence of multifunctional, easy‐to‐adjust sensors makes this technology convenient to use, cost‐effective, and reliable for clinical research (Table 3).

**TABLE 3:** The types of sensors appearing in the microfluidic chip of the urinary system.

Sensor	Principle	Target	Ref.
Electrical signal sensor	Individual cells can be encapsulated and captured by droplets and microelectrodes, and the impedance of different cancer cells is different.	BCa cell	2022 Fan [79]
Image signal sensor	The target cells, after color development, are transmitted to the smartphone mobile device for counting.	Urinary exfoliated tumor cells	2018 Liang [62]
	By controlling different flow rates, the obtained high‐speed camera images are analyzed, and then the mechanical strength of the sample cells is determined by the formula. Finally, the epithelial tumor cancer cells in different periods can be distinguished.	Epithelial tumor cancer cells	2019 Liu [105]

## 3 Microfluidics and BCa

BCa is reported as the most common type of cancer among all urinary neoplasms. Most patients with BCa had an initial diagnosis of non‐muscle invasive BCa [54]. The risk of recurrence of BCa ranges from 30% to 80%, with a 15% chance of developing muscle‐invasive BCa within a few years [54]. Therefore, patients with BCa need to be monitored for an extended period of time to track its progress. The most popular clinical method for BCa evaluation is an invasive examination [55]. Due to the different conditions of patients, a more effective and non‐invasive examination method for BCa is urgently needed.

### 3.1 Urine Examination With Microfluidics

The clinical diagnosis and follow‐up of BCa include urine exfoliative cytology, color doppler ultrasonography, CT scan, and cystoscopy [56]. Among them, urine exfoliative cytology is a standard clinical method for disease monitoring, although with relatively low efficiency [57]. Since extracellular vesicles (EVs) and other metabolites of bladder mucosa and tumors enter the urine through various pathways, they are suggested as potential clinical biomarkers in urine.

#### 3.1.1 Urinary Exfoliated Tumor Cells (UETCs)

UETCs are derived from the cells shed off from bladder tumors and are expected to share the same genomic information with BCa cells [58]. UETCs have been a diagnostic marker in urinary cytology for over 2 decades. Nevertheless, current methods' low sensitivity and poor qualification nature (i.e., lack of assay standardization and inconsistent cut‐off values) have resulted in various limitations in clinical practice [59]. Those defects significantly discouraged the application of UETCs as a biomarker for BCa diagnosis. With a high recurrent rate in BCa, traditional urinary cytology testing is not beneficial in decreasing the screening workload for detecting BCa, especially for patients with recurrent disease. To tackle these issues, novel methodologies were developed to capture UETCs. In 2013, Birkhahn et al. [60] designed a device using polycarbonate membranes with uniformly distributed perforations of 7.5 μm size to capture different types of UETCs. It employs a cell size‐based capture mechanism with a dimension of 7.5 μm comparable to the size of unwanted blood cells in the assay, in which more giant tumor cells are captured and enriched on a reduced surface area. In contrast, smaller blood cells pass through a microfilter. While the specificities of microfilter‐based and conventional cytology were similar (100% vs. 95.8%, *N* = 24), the former's sensitivity increased significantly compared to the latter (53.3% vs. 40%, *N* = 30). One major disadvantage, though, is that the device could be clogged by accumulated debris (both cellular and non‐cellular) and consequently contaminate or even damage the captured cells. To solve this issue, in 2018, Chen et al. analyzed the sizes and distributions of UETCs in urine samples and made a microfluidic device to capture UETCs based on three common sizes (15, 30, and 60 μm) of UETCs that were commonly captured [61]. The microunits with those distinct sizes are collectively grouped in repeat patterns throughout the chamber and allow the capture of single UETCs of various sizes or clustered UETCs. With this modification, an increased cell recovery rate (80.6%) was achieved in cell loss analysis compared to that of the traditional pap smear (71.4%); a numerical representation of cell loss is provided by the RF, which is calculated as follows: RF = ([recovered cells on chip or on slide]/[total input cells] 100%). Increased UETC counts in patients with BCa (*N* = 79) were observed compared to the normal controls (NC) group (*N* = 43) with statistical significance (53.3 [10.7–1001.9] versus 0.0 [0–0.3.0] UETCs/10 mL; *p* < 0.0001). The performance from parallel experiments showed that the sensitivity of microfluidic immunoassay is 95% (*N* = 20), which is much higher compared to traditional cytology and two commercially available FDA‐approved markers (BTA stat and NMP22 Bladder Chek) (35%, 65%, and 35%, respectively). The specificity is comparable to the other three assays (80% for microfluidic immunoassay, 100%, 60%, and 80% for traditional cytology, BTA stat, and NMP22 Bladder Check, *N* = 20). Overall, this experiment demonstrated that UETCs captured by the microfluidic device showed advantages over conventional cytology and two commercially available FDA‐approved markers [61].

**FIGURE 3:**
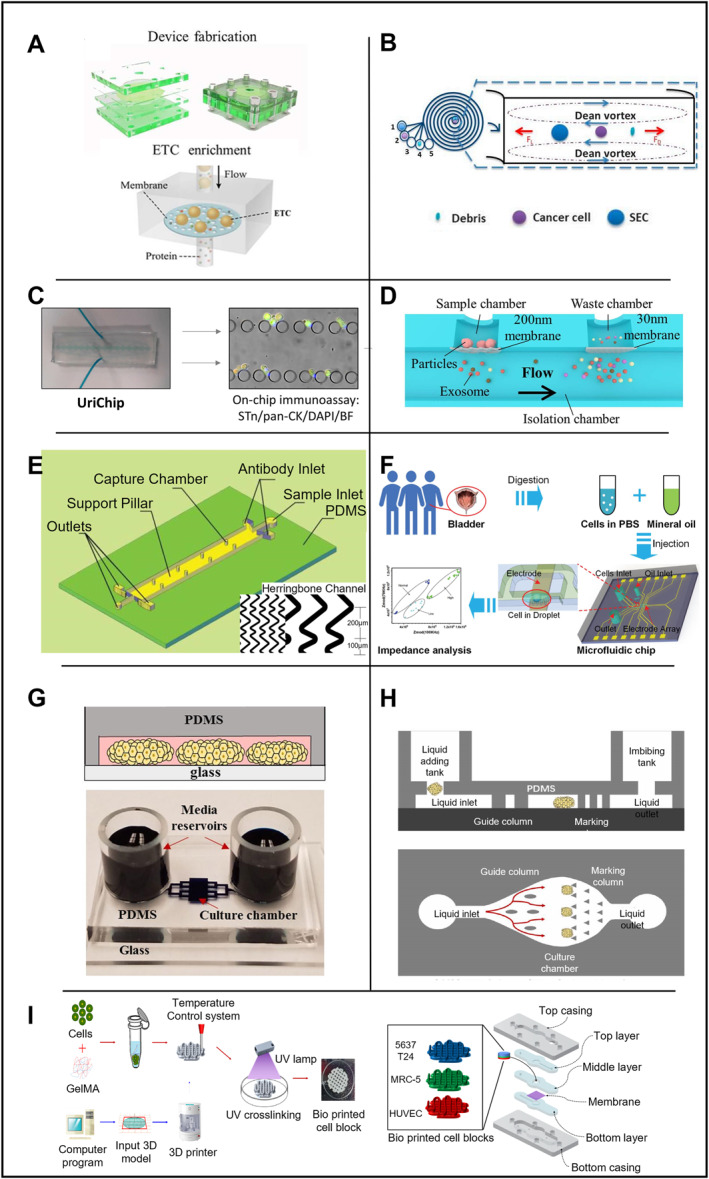
Application of microfluidic technology in bladder organs. (A) Microfluidic device for filtering ETCs by controlling pore size. Reproduced with permission [62]. Copyright 2018, Elsevier. (B) Microfluidic device with spiral channel filtration system. Reproduced under terms of the CC‐BY license [134]. Copyright 2019, The Authors, published by MDPI. (C) A microfluidic chip capable of concentrating fresh or frozen urine for dual biomarker immunoassays such as pan‐CK (epithelial biomarker), Sialyl‐Tn (tumor‐associated biomarker), and DAPI (nuclear staining). Reproduced under terms of the CC‐BY license [65]. Copyright 2020, The Authors, published by Frontiers Media S.A. (D) Dual‐filtration microfluidic device for capturing exosomes. Reproduced under terms of the CC‐BY license [74]. Copyright 2017, The Authors, published by Springer Nature. (E) Herringbone‐structured microfluidic chip for capturing CTCs. Reproduced with permission [77]. Copyright 2020, Taylor & Francis. (F) Impedance‐based microfluidic chip for identifying BCa cells grading BCa OOC. Reproduced with permission [79]. Copyright 2022, Royal Society of Chemistry. (G) BCa cultures in microfluidic devices. Reproduced under terms of the CC‐BY license [84]. Copyright 2017, The Authors, published by Springer Nature. (H) BCa organ‐on‐chip. Reproduced under terms of the CC‐BY license [85]. Copyright 2024, The Authors, published by John Wiley and Sons. (I) Structure of BCa OOC based on BCG vaccine. Reproduced under terms of the CC‐BY license [87]. Copyright 2021, The Authors, published by MDPI.

In 2018, Liang et al. developed a microfluidic integrated device that can separate and concentrate UETCs, as shown in Figure 3A [62]. This device is designed based on size‐exclusion principles with a polycarbonate membrane with a uniform micro‐pore size of 5 μm. UETCs with a diameter > 5 μm are captured for enrichment by the microfluidic device at the fluidic speed of 1 mL/min. The experiment used discarded urine samples, including 20 samples from healthy controls and 35 samples from BCa patients. According to the ROC‐10 curve, when the specificity was 90%, the sensitivity of integrated filter microfluidic devices for BCa diagnosis was 77.1%. One of the device's characteristics is that it has higher integration and is suitable for rapid detection scenarios of POC. After separation and enrichment, exfoliated tumor cells (ETCs) are quantified by direct microchip Enzyme‐Linked Immunosorbent Assay (ELISA). Isolated ETCs bind to anti‐EGFR antibodies coupled to streptavidin‐HRP. After incubation with 3,3′,5,5′‐Tetramethylbenzidine, blue fluorescent signals were formed and captured using a mobile device, and the images were sent to a computer for data analysis. This method does not require centrifugation and traditional climbing analysis of the sample, and it is more convenient to transmit the color image directly to the computer, which is a trend in line with POC. However, the possibility of specific blockage issues cannot be ruled out. Although blood cells of 6–7 μm can be deformed through a small pore size, it is still more sensitive than the 7.5 μm device (77.1% > 53.3%). Therefore, integrated filtering devices have great potential in BCa screening and subsequent analysis of treatment effects.

In 2019, Khoo et al. developed a sorting device for exfoliated BCa cells (EBCCs), which can separate different epithelial‐mesenchymal transition (EMT) phenotypes. Unlike physical size filtration and partition, this study relies on the fluid to sort different subtypes of cells. This program integrates microfluidic analysis based on the principle of inertial focusing, as shown in Figure 3B, for high‐throughput isolation of cell size‐based EBCCs subtype. It works mainly based on the centrifugal force of cells in the fluid cavity. When cells of different sizes flow in the microchannel, they will be concentrated at different positions in the transverse plane [63]. The device can quickly process clinically relevant urine sample volume (20 mL urine in 20 min). The experiment proved that the targeted EBCCs could reach 93.3% recovery rate. Since mesenchymal and epithelial cells have different shapes and deformation characteristics [64], the device can separate different subtypes of epithelial cells and mesenchymal cells into the corresponding outlet of the device. The antibodies used to isolate the biomarkers by this cell population are closely related to BCa, including anti‐survivin antibodies and EGFR. Besides, EBCCs could express a variety of additional biomarkers based on their EMT status, including cytokeratin (CK) and vimentin (VIM), two protein markers with an essential role in the development and progression of Bca, and are considered to have great potential to be utilized in future clinical devices.

In addition to using the above biomarkers, glycan Sialyl Thomsen‐Friedenreich Antigen (Sialyl‐Tn) is also a biomarker for BCa detection. In 2020, Carvalho et al. developed a BCa detection device, UriChip, in which a microfluidic system can enrich and concentrate UETCs either in fresh urine or frozen urine [65] as shown in Figure 3C. Five rows of microcolumns with various column spacings make up the device's core structure, which filters and enriches cells in the urine. UriChip showed a 53.3% capture efficiency for Sialyl‐Tn‐specific UETCs. According to an in situ immunoassay, BCa patients had more sialic acid‐Tn‐positive cells in their urine than healthy controls (*p* = 0.0006). Notably, this is the first instance in which microfluidic technology has been used to combine Sialyl‐Tn levels with urothelial exfoliated cells isolated from BC patients' urine. The work also showed that the contents of UETCs expressing a significant quantity of sialic acid‐Tn may be extracted using frozen urine sediments. The evaluation of cryopreserved urine deposits by microfluidics is among the very first attempts. In conclusion, UriChip employs Sialyl‐Tn as a biomarker to collect UETCs for tumor grading and staging in combination with the microfluidic device, offering a novel method for identifying urothelial BCa cells.

#### 3.1.2 EVs Capture With Microfluidics

EVs belong to a diverse class of membrane‐enclosed vesicles that carry a variety of biomolecules, including lipids, proteins, RNA, and DNA [66]. EVs could be isolated from multiple body fluids, including plasma, urine, saliva, and cerebrospinal fluid, and have distinct physicochemical characteristics highly related to cancer [67]. EVs exchange cellular components as part of intercellular communication to facilitate molecular signaling [68]. Beyond this physiological purpose, mounting evidence indicates that EVs contribute to carcinogenesis by stimulating tumor cell proliferation, augmenting angiogenesis, and increasing invasiveness and migration [69]. Several studies have investigated the composition of EVs qualitatively, particularly in terms of lncRNAs, miRNAs, and proteins. Heat shock protein 90 (HSP90), SDC1, myristoylated alanine‐rich C‐kinase substrate (MARCKS), MARCKS‐related protein (MARCKSL1), tight junction protein 2 (TJP2), and complement decay‐accelerating factor (CD55) were found to be significantly (*p* < 0.05) upregulated in urinary EVs from patients with BCa compared with that in EVs from healthy individuals [70]. Ultracentrifugation is one of the traditional EV extraction methods, yet the whole process is highly dependent on the quality of specific equipment, and time consuming [71]. Microfluidics based on ExoChips [72] and PDMS equipment [73] could separate EVs from serum or plasma. The EVs will then be fluorescently labeled, and the biochemical parameters will be analyzed and compared between cancer patients and healthy controls. In short, EVs can be isolated from microfluidic devices and used in cancer‐related clinical research, providing a novel alternative method to study urinary system‐related cancers.

In 2017, a microfluidic device with a unique double filter structure was generated by Liang et al. [74], as shown in Figure 3D, which is able to filter and screen extracellular vesicles in urine. The corresponding pore sizes are 200 and 30 nm for the two layers of polycarbonate membrane filters. Extracellular vesicles within this size range could be captured and enriched with an efficient capture rate of 74.2%. In the follow‐up microchip quantification analysis by ELISA, sensitivity of 81.3% and specificity of 90% were achieved in 16 patients with BCa and eight healthy controls. Costly high‐speed centrifuges or antibodies which are required using traditional methods are replaced by simple filtering based on physical parameters in those devices. With decreased cost and increased convenience in manufacturing, such devices have great potential in POC as well as serve as a non‐invasive method to assist cancer screening (e.g., BCa).

### 3.2 Circulating Tumor Cells (CTCs) Detection With Microfluidics

Circulating tumor cells (CTCs) are crucial for BCa detection and staging [75]. Studies using CTCs to facilitate cancer detection have grown tremendously in recent years. However, how to capture circulating tumor cells economically and efficiently is still an open question [76]. In 2020, Wang et al. created a microfluidic tool with an innovative herringbone channel structure to collect CTCs, as shown in Figure 3E. Monoclonal antibody BCMab1 against BCa was modified with biotin prior to the experiments, and human BCa cells (T24) were spiked into blood specimens from healthy volunteers to mimic circulating tumor cells in patients. The staggered herringbone pattern produces a capture efficiency of 90% (vs. a 49% capture efficiency using chips without a staggered herringbone pattern) with high specificity [77]. The capture rate of CTCs was 90% at a cell concentration of 5000 cells/mL, which improved significantly compared to the efficiency of CellSearch (28.3%) [78]. A wider groove with increased groove pitch is also suggested to improve cell capture [75]. Furthermore, since each streptavidin molecule can bind to up to four biotin molecules, they developed a multi‐level signal amplifier system to maximize the signal [78]. In summary, this system could achieve increased cell capture yields, efficiency, stability and specificity.

In 2022, researchers developed an impedance‐based microfluidic chip [79], as shown in Figure 3F. They generated microdroplets at the single‐cell level using cancer cell lines and cells from clinical samples and introduced an integrated microfluidic electronic sensor to calculate the grade of BCa. Droplet microfluidics uses compartments that are on the same size scale as the cell, and it could combine with high‐accuracy, high‐sensitivity droplet analysis methods, such as fluorescence detection, mass spectrometry, electrochemical detection and surface‐enhanced Raman scattering for single‐cell analysis. The electrical properties of cells could facilitate the understanding of the complex physiological states of cells and help classify various types of tumor cells [80], stem cells [81], and blood cells [82] without specific labeling during the detection by electronic sensors. When it comes to BCa cell categorization, the electrical detection system can distinguish between tumor samples and normal epithelial tissue using droplets and microelectrodes [80, 83]. Individual cells may be wrapped and captured by these droplets and microelectrodes, and their impedance can be measured in a label‐free and non‐invasive pattern. The classification of cancer cells was conducted according to the impedance of different measurements.

### 3.3 BCa OOC Application

Patient‐derived xenografts (PDXs) are one of the most commonly used in vivo models for drug screening. PDX models are generated by engrafting human cancer cells into immunodeficient mice. PDXs have been generated for pancreatic cancer, hepatocellular carcinoma, breast cancer, PCa, and many other cancer types with promising clinical benefits. However, the high cost, low efficiency of engraftment, and long development time increase the financial burden of patients and have limited its usage to a small scale for clinical applications. Researchers have been looking for low‐cost replacement of PDX models, such as microfluidic devices that maintain BCa cells over extended periods to study patterns of drug responsiveness and possible resistance [84]. As shown in Figure 3G, by culturing PDX and clinical patient specimens, the authors confirmed that the microchambers could maintain the phenotype of primary cancer cells long enough to evaluate cancer drug responsiveness and resistance. Also, they demonstrated that microchamber cultures from specific PDX models retained patterns of drug responsiveness and resistance observed in mice by a proof‐of‐concept experiment. This result is encouraging and could pave the way to a reliable microfluidics‐based platform for the long‐term cultivation of cancer cells and screening of anti‐cancer drugs.

In 2023, Xiong et al. [85] designed a high‐throughput OOC for the treatment of BCa (Figure 3H). The experimenters first cultured tumor organoids in vitro, then constructed a microfluidic platform and finally combined the organoids with the microfluidic platform to establish an OOC system for drug testing. The results showed that the organoids cultured in vitro were genetically similar to the tumors of primary patients and maintained the essential characteristics of in situ tumors. The microfluidic chip has three layers (upper, middle, and lower). The upper layer is a thick PDMS membrane structure with two holes at the inlet and outlet to add and remove culture media and drugs. Then, the organoid suspension was injected into the chip with a pre‐added medium for culture. A critical design feature involved streamlined guiding pillars within the microfluidic channel, which achieved three essential functions: flow rate modulation, bubble suppression, and spatial segregation of organoid spheroids into discrete chambers. Comparative validation demonstrated a strong correlation between in vitro drug sensitivity profiles (*n* = 8), patient‐derived xenograft (PDX) model responses (*n* = 2), and clinical outcomes in corresponding patients (*n* = 2). These findings collectively indicate that the engineered organ‐on‐a‐chip system can effectively simulate clinical drug responses and validate the differential sensitivity of patient‐specific organoids to selected chemotherapeutic agents. The above results show that the constructed OOC can initially simulate clinical medication and verify the sensitivity of different patient organoids to a selected variety of chemotherapeutic drugs.

*Bacillus* Calmette‐Guerin (BCG) is a common intravesical immunotherapy drug used in patients with BCa. BCG stimulates innate immune and inflammatory cancer responses to eliminate BCa cells [86]. In 2021, starting with immunotherapy, Kim et al. constructed a BCa OOC built upon BCG [87] capable of simulating the tumor microenvironment (TME) in vitro, as shown in Figure 3I. In this experiment, 3D printing technology was used to combine cells with gelatin methacryloyl (GelMA) hydrogel to print into cell blocks. The cell source was based on T24 and 5637 cell lines, including MRC‐5, HUVEC, and THP‐1 cells. Then, the printed cell blocks were packaged into a microfluidic chip. The results showed that the cells in the round cell block could remain alive under the condition of 20 μL/min microfluidic flow and 15% cell filling density in the hydrogel. The cell migration increased with the BCG dosage, while cancer cell proliferation was inhibited with the increase of BCG concentration. This study assessed the cell survival and proliferation rate, chemotaxis of monocytic THP‐1 cells, and concentration of cytokines following treatment with BCG in order to determine the immunologic effects of BCG in BCa OOC (BCOC). The cell viability of BCOCs decreased in a dose‐dependent manner after BCG treatment and was statistically significant in the 5637 BCOCs after BCG treatment at 1, 10, and 30 MOI (59.4 ± 1.3, *p* < 0.05; 52.7 ± 1.0, *p* < 0.05; and 20.6 1.3, *p* < 0.01, respectively). Based on these findings, the chip can potentially facilitate immunotherapy for BCa by determining the dosage of BCG in patients. Due to the use of cell lines and hydrogels used in common studies as substrates, this study did not simulate the specific cells of real individuals' blood and urine environment, and further studies are required to justify such simulations in vitro (Table 4).

**TABLE 4:** Summary of the application of microfluidic technology in bladder organs.

Years author	Target for testing	Microfluidic method	Experiment purpose
2017 Liang [74]	Urinary extracellular vesicles (EV)	Based on the physical barrier, 30–200 nm EVS was separated and concentrated from urine by a microfluidic device, and then EVS was quantified by microchip ELISA.	Quantitative filtration and screening of urinary extracellular vesicles in urine.
2018 Chen [61]	Two oncoproteins CK20 and CD44v6 Antigens in Urine Exfoliated Tumor Cells (UETCs)	The microfluidic device captures intact exfoliated urine tumor cells (UETCs) individually or in groups.	A microfluidic device for collecting and identifying urine‐exfoliated tumor cells (UETCs)
2018 Liang [62]	BC4 exfoliated cells (ETCs)	An integrated filter with an aperture of 5 microns to collect ETCs	ETCs were separated and concentrated in urine samples and then quantified using a microchip ELISA.
2019 Khoo [134]	Invasive mesenchymal exfoliated BCa cells (EBCC)/epithelial‐mesenchymal transition (EMT) phenotype cells	High‐throughput separation of EBCC subtypes based on cell size using a microfluidic device integrated with inertial focusing	An invasive mesenchymal exfoliated BCa cells (EBCCs) sorting (ES) device can be used to isolate malignant EBCCs for real‐time detection and isolation of the biomarker within hours.
2020 Carvalho [65, 77]	Urinary epithelial exfoliated tumor cells/cancer‐associated dextran sialyl‐TN	Concentrate urothelial exfoliated cells in fresh and frozen urine using a microfluidic‐based UriChip device.	Isolation of urothelial exfoliated cells from cryopreserved urinary sediment
2020 Wang [77]	Circulating BCa cells based on antibody‐BCMab1	A capture platform consisting of a polydimethylsiloxane (PDMS) chip was designed. The team also introduced herringbone or herringbone channel patterns into the chip.	Specific capture of circulating tumor cells from BCa patients for detection and treatment of BCa
2022 Fan [79]	Human BCa cell	Using the principle that different cells have different impedances under the action of electrodes, their impedances are measured in a label‐free and non‐invasive manner.	Grading of BCa cells
2017 Gheibi [84]	Human BCa cell	Development of an in vitro microfluidic culture platform for studying drug reactivity and resistance patterns	Drug responsiveness and drug resistance in an in vitro model of BCa cells
2023 Xiong [85]	BCa tissue samples	Through the further study of organoid chips for BCa, a simple, efficient, high‐throughput organoid chip for the vast majority of BCa patients undergoing chemotherapy was constructed	Construction of a clinical platform for individualized treatment of BCa
2021 Kim [87]	Based on T24 and 5637 cell lines, MRC‐5, HUVEC and THP‐1 cells	Construction of Tumor Microenvironment TME using 3D bioprinting and microfluidic technology for immunotherapy of BCa	Development of a tumor microenvironment (TME) BCa chip (BCOC) that mimics immunotherapy in vitro)

## 4 Microfluidics and PCa

PCa is a prevalent malignancy in males worldwide [88]. The prognosis of PCa confronted many challenges. In this section, we will focus on prostate‐specific antigen (PSA), exosome detection, PCa staging, and the detection of prostate‐circulating tumor cells.

### 4.1 PSA Detection With Microfluidics

PSA is an efficient PCa biomarker commonly used in PCa screening [3]. PSA exists in both free status and bound with other proteins in the blood. However, traditional PSA tests have disadvantages, such as high sample consumption and restricted analyte control in the reaction chamber. Using microfluidic devices is thus expected as an effective method to overcome these deficiencies. Currently, automated analyzers are used in centralized facilities with high throughput for PSA testing. This procedure is time‐consuming and needs extensive infrastructure, including strict sample transportation regulation and professionally trained medical personnel. In 2019, Maj‐Hes et al. [89] developed a microfluidic‐based fingertip blood PSA rapid quantitative analysis system named Claros. Claros analyzer consists of a disposable microfluidic test cartridge (approximately the size of a credit card) and a compact analyzer (12 × 9 × 20 cm). The assay is founded on the principle of a silver amplification immunoassay. The sample and reagents travel through the 50‐micron microfluidic channels during the analysis. Antigens of interest (PSA) are captured in predetermined regions (detection zones) of the channels, which apply microfluidic technology to perform POC detection directly from fingertip blood. In the experiment, the researchers compared the PSA levels in the experimental samples (100 males asymptomatic for prostate disorders) with the data from two commercially available PSA tests conducted by a reference laboratory (Abbott and Elecsys by Roche). The results showed a high correlation, indicating that Claros has the advantage of instant detection while ensuring effectiveness.

**FIGURE 4:**
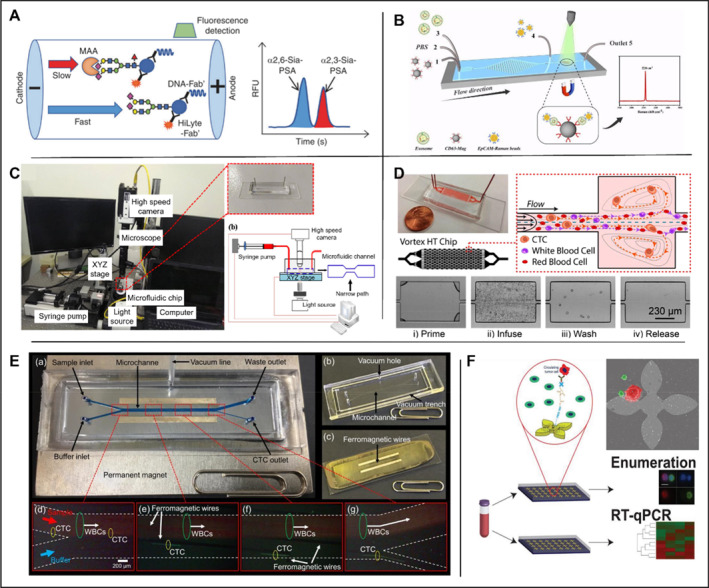
Application of microfluidic technology in prostate organ. (A) The α2,3‐Sia‐PSA assay system. MAA was included in the buffer to separate α2,3‐Sia‐PSA from α2,6‐Sia‐PSA by affinity electrophoresis. Fluorescence signals from laser‐induced fluorescence detection were analyzed, and the percentage of α2,3‐Sia‐PSA was calculated from their peak areas. Reproduced with permission [96]. Copyright 2022, The Authors, published by Springer Nature. (B) Exosome detection and capture using a microfluidic Raman chip. Reproduced with permission [102]. Copyright 2020, Royal Society of Chemistry. (C) Based on the analysis of different cancer cells. Reproduced under terms of the CC‐BY license [105]. Copyright 2019, The Authors, published by MDPI. (D) A vortex microfluidic chip for isolation of prostate CTCs. Reproduced under terms of the CC‐BY license [108]. Copyright 2017, The Authors, published by Springer Nature. (E) A lateral magnetophoretic microseparator for separation of CTCs for a deeper understanding of cancer characterization. Reproduced under terms of the CC‐BY license [110]. Copyright 2020, The Authors, published by MDPI. (F) A microfluidic device (GO chip) based on graphene oxide for circulating tumor cell counts and RNA extraction, respectively. Reproduced under terms of the CC‐BY license [112]. Copyright 2019, The Authors, published by John Wiley and Sons.

Total PSA, free PSA, and intact PSA have all demonstrated reasonably strong predictive effects in PCa [90]. However, PSA screening based on concentration alone has been linked to an increase in low‐grade PCa diagnosis, which has an almost 100% 5‐year survival rate [91]. Due to the lack of specificity for high‐grade PCa, positive PSA screening results often result in overdiagnosis, overtreatment, and unnecessary follow‐up operations [92]. Thus, there is an urge demand for PCa screening with higher specificity of high‐grade PCa, which has a much worse prognosis than low‐grade PCa. Increasing evidence suggests that patients with PCa have higher levels of α‐2,3 sialylation and fucosylation than those with benign prostatic hyperplasia [93]. In addition, differentiating between high‐grade and low‐grade PCa using changed PSA glycosylation patterns may benefit patients with PCa [94]. Since glycoprotein sialylation and fucosylation are differentially regulated [95], a combination analysis of serum PSA fucosylation and 2,3‐sialylation will likely increase the specific detection of high‐grade PCa. In 2022, Hatano et al. [96] used microfluidic electrophoresis to measure α2,3‐Sia‐PSA and α‐1,6‐Fuc‐PSA using a microfluidic immunoassay system [97]. 2,3‐Sia‐PSA and 1,6‐Fuc‐PSA concentrations were measured using a microfluidic immunoassay system based on the liquid‐phase binding electrokinetic analyte transport assay principle [97, 98]. In the 2,3‐Sia‐PSA assay instrument, fluorescence signals from laser‐induced fluorescence detection were analyzed by software designed to use internal fluorescent markers to align and identify the peaks of 2,3‐Sia‐PSA and 2,6‐Sia‐PSA (Figure 4A). The serum samples were thawed and simultaneously analyzed. The time between blood collection and storage in the freezer was approximately 3 h. In the α2,3‐SiA‐PSA detection system, the peaks of α2,3‐SiA‐PSA and α2,6‐SiA‐PSA were detected and analyzed based on laser‐induced fluorescence. Their data suggested that the percentage of α2,3‐Sia‐PSA and the percentage of α1,6‐Fuc‐PSA were significantly higher in patients with high‐grade PCa than in those with negative biopsies or with low‐grade PCa (*p* < 0.0001). These findings suggest that a combination model of 2,3‐Sia‐PSA and 1,6‐Fuc‐PSA could effectively identify patients with high‐grade PCa and distinguish them from those with low‐grade PCa or from health controls. Large‐scale prospective studies are required to validate these findings.

### 4.2 Microfluidics and PCa Exosome Detection

Exosomes, with diameters ranging from 30 to 200 nm, can be produced in multiple kinds of body fluids such as blood, saliva, and urine [99]. Exosomes' molecular contents represent the particular physiological circumstances and roles of their original cells [100]. Due to their high concentration and steady circulation status, tumor‐derived exosomes are considered as viable liquid biopsy biomarkers for cancer patients. Most approaches to isolate exosomes depend on general physicochemical characteristics, such as particle size and density. The most popular technique for exosome isolation is ultracentrifugation, which requires long‐time centrifugation (> 8 h), but the yield and purity are usually poor [101]. A microfluidic Raman biochip for in situ exosome extraction and analysis was created by Wang et al. in 2020 [102] as shown in Figure 4B. CD63 antibody is used for the capture of exosomes due to its high expression in exosomes and EpCAM bound Raman beads were used to detect PCa specific cells due to the significantly different expression levels of EpCAM in exosomes from PCa cells and normal cells. The procedure is designed as follows: firstly, anti‐CD63 magnetic nanoparticles (Mag‐CD63) that can secure exosomes in a particular position are created. Secondly, an EpCAM‐Raman bead with a high density (EpCAM‐Raman pearls) was created to facilitate signal detection. The specific principle is depicted in the diagram: Mag‐CD63 and the detected exosomes were injected into the first and third ports, respectively, and thoroughly mingled through a rectangular structure; Phosphate buffered saline (PBS) was administered through the second port. EpCAM‐Raman beads were rinsed and injected into the fourth port to blend them in the subsequent circular Raman detection area, and washed by PBS again. 20 μL of exosome samples could be analyzed per hour by Raman spectroscopy. The analysis of clinical samples verified that more exosomes were isolated from the serum of PCa patients compared to that of healthy individuals. The average number of exosomes in the serum of PCa patients was calculated to be 10.61 × 10^8^ particles/mL, approximately three times that of healthy controls. The authors conclude that microfluidic Raman devices can effectively differentiate between PCa patients and healthy controls (*p* < 0.0001).

### 4.3 Microfluidics and PCa Cell Staging

The initial development of PCa is often androgen‐dependent, so androgen deprivation therapy (ADT) has always been the standard care and first‐line therapy for androgen‐dependent PCa. Unfortunately, most PCa patients eventually relapse into the androgen‐independent growth stage, which is resistant to ADT and usually associated with poor prognosis [103]. Since cancer migration and invasion potential is highly correlated with their mechanical strengths, it was suggested that measuring the mechanical strength for sample cells might be indicative of their metastatic potential [104]. In 2019, Liu et al. conducted morphological‐rheological microfluidic‐based high‐throughput mechanical phenotyping of androgen‐sensitive and insensitive human PCa cell lines [105]. Although this morphological rheology has been studied concerning blood cells, it is yet unknown how it will be employed in epithelial carcinoma cells as shown in Figure 4C. In this study, a high‐speed camera was combined with a microfluidic chip of 25 × 25 × 300 μm (width × height × length). Images were captured and analyzed under different flow speeds, and the mechanical strength of the sample cells was calculated. Thus, the androgen‐sensitive epithelial tumor cells (LNCaP, DU145, and PC3) at different stages can be distinguished. The results showed that the elastic modulus of LNCaP cells, DU145 cells and PC3 cells were 1.08 kPa, 1.44–2.4 kPa, and 1.87–2.40 kPa, respectively, suggesting significantly different mechanical features of those cells. The mechanical strength of LNCaP cells was the lowest, followed by DU145 cells, and the mechanical strength of PC3 cells was the highest, supported by the measurements by Atomic Force Microscopy (AFM). Therefore, compared with ADT, this technique has a potential for early detection of PCa based on mechanical strength. Compared to the high cost associated with using an atomic force microscope, using microfluidic technology and a high‐speed camera lowers the cost of mechanical characterization of cancer cells.

### 4.4 Detection of Prostate CTCs

Non‐invasive liquid biopsies have become more prevalent for early cancer detection, monitoring and prediction of drug responses in recent years. CTCs have emerged as biomarkers to provide genetic and phenotypic information during cancer evolution from primary sites to metastatic sites [106]. Current separation methods are based on the size of different cells in the blood [107] yet the purity of separated CTCs and operation simplicity are limited. In 2017, Renier et al. designed a device that uses an eddy current microfluidic chip to isolate CTCs from the blood of PCa patients [108], as shown in Figure 4D. The device tracks the movement of cells in the microfluidic channel to screen and enrich relatively large cells in a high‐speed flowing fluid (8 mL/min). In 2019, Obayashi et al. updated the microfluidic device with lower cost and better sensitivity, and the chip is composed of two different types of micropillar arrays. T. Ohnaga et al. [109] further modified the device to prevent whole blood from hindering the channels. The distance between micro‐posts was increased to 200 mm near the chip inlet. The device was coated with mouse anti‐human EpCAM antibody (primary) and goat anti‐mouse IgG antibody (secondary). The polymer CTC‐chip immobilized with surface antibody was placed in a holder that allowed a liquid sample to flow through a channel. The two apertures of the holder were then connected to a syringe pump and a sample tube. The capture efficacy was determined by comparing the number of cells remaining on the chip after sample passage to the number of cells that entered the chip inlet. To count CTCs in patients with metastatic PCa, they used a CTC‐capture polymer chip. The average capture rate of PC3 cells in PBS was 94.60%, and the average capture rate in whole blood was 83.82%. The average capture rate of LNCaP cells in PBS was 82.73%, and the average capture rate in whole blood was 75.78%. In 2020, Cho et al. [110] created a lateral magnetophoretic microseparator for CTC separation to better understand the malignancy features, as shown in Figure 4E. The performance was compared between the lateral magnetic induction micro‐separator (“CTC‐µChip”) and the commercially available specialized method AdnaTest ProstateCancer (Qiagen) for the isolation of CTCs from the blood of PCa patients. The results showed that CTC was detected in 14/14 (100%) patients using CTC‐μChip and 9/10 (90%) patients using AdnaTest. The average CTCs separated by CTC‐μChip and AdnaTest were 14.8 and 0.83 CTC/mL, respectively. The contrast is even sharper in samples from patients with metastatic PCa, with 20.54 versus 2.29 CTCs/mL by CTC‐μChip and AdnaTest, respectively. In conclusion, the average number of CTCs isolated by CTC‐μChip was higher than that of CTCs isolated by AdnaTest.

Real‐time, non‐invasive methods for genomic analysis of tumors are of specific interest largely due to the highly variable disease progression and treatment response in advanced PCa. Due to their propensity to be informative through enumeration and RNA expression, CTCs have a unique potential as therapeutically effective biomarkers in liquid biopsy. Kozminsky et al. used the microfluidic chip, which was originally developed by Yoon et al., to study prostate CTCs [111]. The captured CTC extracted RNA from the parallel microfluidic chip, as shown in Figure 4F, to determine the CTC features associated with the progression and survival of advanced PCa. CTCs were isolated from the whole blood of healthy individuals (*n* = 8) and patients with metastatic castration‐resistant PCa (*n* = 41). The median CTC count was 20 CTC/mL in PCa samples (3‐166 CTC/mL), and that in healthy controls was 3 CTC/mL (0‐14 CTC/mL), with a statistically significant difference (*p* = 0.0001). This study demonstrates that the CTC separation device can continue to conduct in‐depth analysis of the patient's CTC in combination with single‐cell techniques such as sensitivity‐based RT‐qPCR technology [112] (Table 5).

**TABLE 5:** Summary of the application of microfluidic technology in prostate organs.

Years author	Target for testing	Microfluidic method	Experiment purpose
2019 Liu [105]	LNCaP, DU145 and PC3 PCa cell lines with different androgen sensitivity	Based on the morphorheological microfluidic method, the mechanical properties of LNCaP, DU145 and PC3 PCa cell lines with different androgen sensitivity were measured quantitatively.	High throughput mechanical phenotype identification of androgen sensitive and androgen insensitive human PCa cell lines
2017 Renier [108]	Prostate circulating tumor cells (CTCs)	Separation of CTC from the blood of patients with PCa by eddy current microfluidic chip	Separating CTC
2019 Kozminsky [112]	Prostate circulating tumor cells (CTCs)	A GO‐based microfluidic device (GO chip) separates the patient's CTC and then detects the expression of related genes by RT‐qPCR	Separation of CTC and CTC clusters
2019 Obayashi [135]	Prostate circulating tumor cells (CTCs)	Using a microfluidic device, a polymer ctc chip immobilized with surface antibodies is placed in a stent, allowing liquid samples to flow through the channel to capture CTCs	Develop a smaller, lower cost CTC capture system
2020 Cho [110]	Prostate circulating tumor cells (CTCs)	A side magnetophoresis microseparator was developed and compared with other commercially available separation devices	Separating CTCs to better understand the characteristics of cancer
2019 Maj‐Hes [89]	Prostate specific antigen	Combined with microfluidic technology, blood can be taken directly from the fingertip for POC detection.	Development of a rapid quantitative analysis system for fingertip blood prostate specific antigen (PSA) based on microfluidic technology
2022 Hatano [96]	α2,3‐Sia‐PSA and α‐1,6‐Fuc‐PSA	Microfluidic electrophoresis was used to determine α2,3‐Sia‐PSA and α‐1,6‐Fuc‐PSA with a microfluidic immunoassay system.	Development of a predictive device for high‐grade PCa
2020 Wang [102]	Exosomes	Using microfluidic technology, exosomes are quantified by enrichment in a microfluidic channel followed by Raman detection of the sample	In situ isolation and analysis of exosomes

## 5 Microfluidics for RCa

RCa or renal cell carcinoma (RCC) is the sixth most frequent cancer and accounts for 2.4% of all cancers in adults [113]. With 71% 5‐year survival rate [114], 30% of patients with RCa had signs of distant metastases at original diagnosis, which usually links to a poor prognosis [114].

### 5.1 RCa Cell‐Free DNA (cfDNA) Detection With Microfluidics

Elevated amounts and fragmentation of circulating cfDNA in plasma have been clinically detectable [115], which has been helpful for the diagnosis, prognosis, and monitoring a range of malignancies [116]. Studies have shown that the length of cfDNA fragments might have a correlation with the progress of cancer [117]. In 2018, Yamamoto et al. [118] took the fragment size of cfDNA into consideration when referring to the relationship between the overall level of cfDNA and RCa. A microfluidic‐based platform was then used to measure the size of cfDNA fragments using plasma samples from RCC patients (*n* = 92) and healthy individuals (*n* = 41). The results of real‐time PCR detection of these samples showed that the plasma cfDNA level of RCC patients was higher, and the fragment size of plasma cfDNA from RCC patients was shorter, when compared to that of healthy controls. However, the two groups had no statistical difference (*p* = 0.052). Interestingly, in patients with hepatocellular carcinoma, the size distribution of plasma cfDNA is transferred to shorter fragments as the proportion of tumor‐derived DNA increases [119]. These studies demonstrate the strong potential of cfDNA fragment size to facilitate RCC diagnosis.

### 5.2 Renal OOC

**FIGURE 5:**
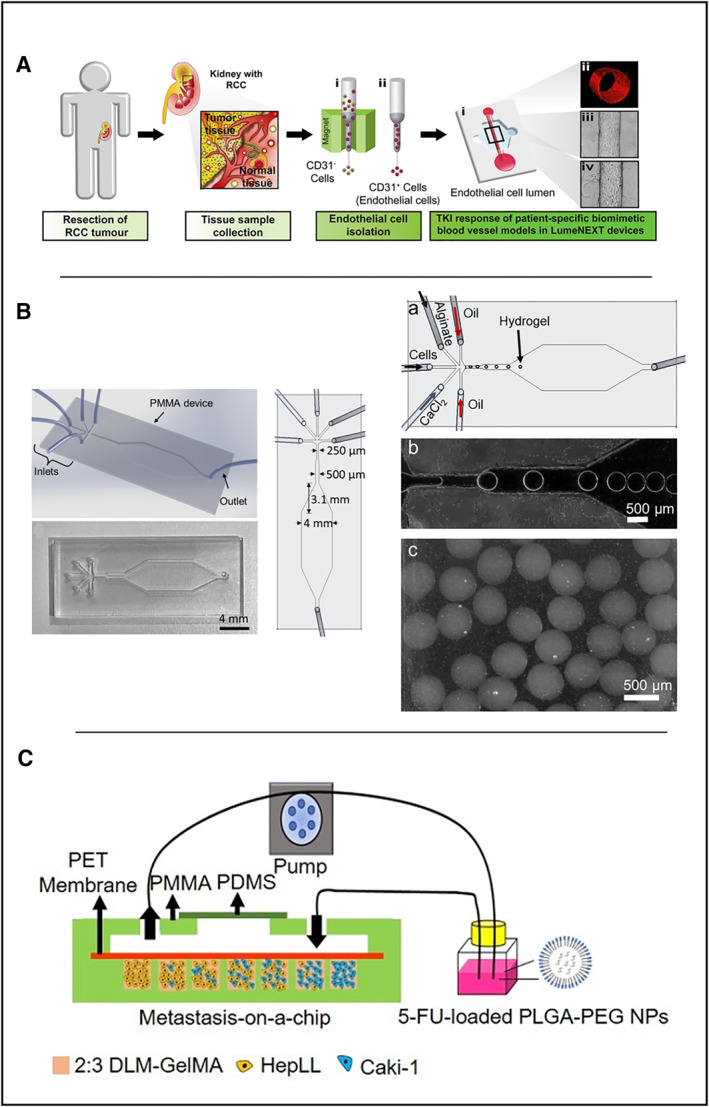
Application of microfluidics in kidney organs. (A) Microfluidics‐based in vitro model in which primary patient‐specific TEnCs and NEnCs are used to create patient‐specific biomimetic blood vasculature. Reproduced with permission [121]. Copyright 2019, The Authors, published by Elsevier. (B) Five‐entry On‐Chip Organoid structure diagram. Reproduced under terms of the CC‐BY license [127]. Copyright 2022, The Authors, published by MDPI. (C) Schematic of the tumor progression model based on metastasis‐on‐a‐chip. Reproduced under terms of the CC‐BY license [130]. Copyright 2020, The Authors, published by Ivyspring International Publisher.

Anti‐angiogenic RCa therapy is an essential means to inhibit tumor growth in cancer treatment. However, optimization of the dosage of anti‐angiogenic drugs is challenging in different patients, and the response to different drugs varies significantly in individuals [120]. Therefore, more efficient in vitro models are required for drug screening studies of patients with RCa. In 2019, Jiménez‐Torres et al. used a microfluidic‐based in vitro model in renal cell carcinoma anti‐angiogenic drug response testing [121] in which normal endothelial cells (NEnC) and primary patient‐specific tumor endothelial cells (TEnCs) were used to create patient‐specific biomimetic blood arteries, as shown in Figure 5A. A LumeNext microdevice method was used to fabricate vascular OOCs in this experiment [122], which is similar to casting technology. The hydrogel is poured into a cavity filled with rod‐like PDMS material, and then solidified completely. After removing the PDMS rod, a hydrogel tubular cavity is obtained. The extracted tumor and regular cell suspensions were added to the microchip for culture. In the subsequent drug response experiments, paired normal and cancer tissues from clear cell renal cell carcinoma (ccRCC) patients (*n* = 5) were combined with microchips to form an in vitro model of patients, and three different concentrations (10 nM, 500 nM, 1 μM) of anti‐angiogenic drugs were used to treat bionic blood vessels [123]. Surprisingly, only some patients' in vitro test models responded to anti‐angiogenesis therapy. This finding is consistent with the clinical observation of response heterogeneity and the development of resistance to anti‐angiogenic therapy [124]. Therefore, these models can potentially evaluate personalized responses to different anti‐angiogenic drugs and facilitate the clinical decisions.

In the drug testing of RCa, the development of organoids has gained great interest due to the urgent need of reliable preclinical models for drug sensitivity and dosage evaluation [125]. However, current patient‐derived organoid models are still insufficient for extensive drug screening. One concern is that the organoid model must be identical in each passage, consistent in size, and able to compare the tested drugs and drug interactions previously shown in primary tumors [126]. To tackle this issue, Ozcelik et al. constructed a microfluidic system to form organoid microspheres with controllable size in alginate hydrogel [127]. This study used a simple and low‐cost microfluidic device to evaluate RCa cells and mesenchymal stem cells (MSC) in drug screening. The kidney‐derived human RCa cells Caki and MSC isolated from human umbilical tissue were combined with sodium alginate to construct RCa cell organoids. A five‐entry microfluidic device was used to prepare organoids as shown in Figure 5B. Alginate, 0.1 M CaCl_2_ solution, mineral oil with 5 wt% SPAN 80, and cell solution were infused from the five inlets of the microfluidic device at 10, 20, 20, 50 and 50 μL/min to form uniform size alginate hydrogel beads containing cells. The organoid beads were placed in the medium for 3D culture. After 21 days, the cell mass was cultured into spheres, and the organoid structure was observed by sectioning. Cisplatin was used as a chemotherapeutic agent for comparison in the drug test. This proof‐of‐concept experiment confirmed that microfluidics and the renal organoid model are suitable for kidney cancer‐related drug testing.

Cancer metastases are responsible for 90% of cancer deaths [128]. Chemotherapy is the major treatment option for patients with metastatic cancer, yet the survival rate still remains low after the treatment [129], which leads to urgent needs for drug testing models for patients with metastatic RCa. In 2020, Wang et al. produced an OOC that can simulate the process of primary RCa cell invading/metastasizing to liver [130]. The chip was based on rat decellularized liver matrix (DLM) and GelMA scaffolds. Seven different proportions of RCa cells (Caki‐1 cells, or C) and hepatocytes (HepLL cells, or H) (100% HepLL; 9:1 H/C; 7:3 H/C; 5:5 H/C; 3:7 H/C; 1:9 H/C and 100% Caki‐1) were co‐cultured in separate chambers, as shown in Figure 5C. The chip material was provided with oxygen permeability by PDMS, and the response to 5‐fluorouracil (5‐FU) on RCa cells was tested. The results showed that 5‐FU‐loaded PLGA‐PEG nanoparticles were significantly more effective than free 5‐FU in removing Caki‐1 cells (*p* < 0.05), indicating that the model is able to predict drug responses and optimize drug dosage on metastatic cancer cells.

Shiga toxin‐producing *Escherichia coli* (STEC) can cause infection in the human intestine, which can be treated by Ciprofloxacin (CIP). However, CIP treatment would increase the secretion of Shiga toxin 2 (Stx2) and result in damage to the human kidneys and lead to hemolytic uremic syndrome (HUS) [131]. To study and observe the phenomenon of infection in vitro, in 2021, a multi‐organ chip combining the intestine and kidney was developed by Lee et al. [132]. It was a gut‐kidney axis (GKA) OOC and used intestinal cells (Caco‐2) and renal cells (HKC‐8). The results showed that the OOC could detect STEC infection and Stx2 secretion/poisonous effect. The chip was used to verify the effectiveness of gentamicin in treating STEC infection and prove that gentamicin can suppress the harmful effect on renal cells during treatment compared to CIP (Table 6).

**TABLE 6:** Summary of the application of microfluidic technology in kidney organs.

Years author	Target for testing	Microfluidic method	Experiment purpose
2018 Yamamot [118]	cfDNA	Measurement of cfDNA fragment size based on microfluidic platform	Relationship between human cfDNA related parameters and renal cell carcinoma
2019 Jiménez‐Torres [121]	Specific tumor endothelial cells (TEnCs) and normal endothelial cells (NEnC)	Patient‐specific biomimetic blood vessels constructed using an in vitro model based on microfluidics	Relationship between antiangiogenic drugs and renal cell carcinoma
2022 Ozcelik [127]	Caki and MSC	Using alginate, CaCl2 solution, mineral oil, and cell solution to form organoid microspheres	Constructed a microfluidic system to form organoid microspheres with controllable size in alginate hydrogel
2020 Wang [130]	Cancer cells (Caki‐1) and hepatocytes (HepLL)	A microfluidic platform was developed in which the medium was connected to two culture zones, and the signal molecules could pass through half a wall (paracrine signal)	Studying the metastasis of RCa cells in hepatocytes
2021 Lee [132]	Intestinal cells (Caco‐2) and renal cells (HKC‐8)	A multi‐organ chip combining the intestine and kidney was developed for the observation and study of OOCs that can observe the mechanisms of STEC infection and STx poisoning.	To study and observe the infection of Shiga toxin‐producing *Escherichia coli* (STEC) in the human body.

## 6 Challenges and Future Perspectives

### 6.1 Limitations

The review summarizes recent studies of microfluidic and OOC technology in urinary tumors. Currently, there are still some limitations that might hinder the application of those technologies in patients with urinary system neoplasms.

Various stages of BCa necessitate distinct treatment approaches. The staging of BCa is closely correlated to circulating bladder tumor cells. The use of microfluidic technology makes it possible to collect BCa CTCs efficiently. It can optimize the study of the correlation between BCa CTCs and staging, combine the different electrical impedances of BCa cells at various stages, and sort cancer cells by combining microfluidic technology with sensors. BCa OOCs maintain the in vitro culture of primary cancer cells or tumor‐like organs, and uncomplicated drug response and immune response experiments can be conducted simultaneously to demonstrate its efficacy. These OOCs are limited in their ability to simulate the tumor microenvironment based on the time and space of cancer cell culture, and there is still much opportunity for improvement. (Tables 7 and 8).

**TABLE 7:** Comparison of microfluidic research with commercial and traditional methods in bladder tumors.

Product or research	Patients or samples	CE (100%)	Targets	Sensitivity	Specificity	Cost/dollar	Ref.
Conventional cytology	Urine of healthy and BC patients	71.4% [61]	UETCs	BC 40% (12/30) [61], BC 35% (7/20) [60]	NC 100% (20/20) [61], NC 95.8% (23/24) [60]	Total cost 11227.63 (6762.95–16565.29)	[60, 61]
BTA	Urine of healthy and BC patients	NR	UETCs	BC 65% (13/20)	NC 80% (16/20)	NR	[61]
NMP22	Urine of healthy and BC patients	NR	UETCs	BC 35% (7/20)	NC 60% (12/20)	NR	[61]
CellSearch	Blood of cancer patients	28.3%	CTCs	28.3% (13/46)	NR	220000 initial cost, 1000/operating cost Obayashi	[78, 135]
2013, Birkhahn et al.	Urine of healthy and BC patients	NR	UETCs	BC 53.3% (16/30)	NC 100% (24/24)	Total cost 136.40 (63.20–309.70)	[60]
2017, Liang et al.	Urine of healthy and BC patients	74.2%	Evs	BC 81.3% (13/16)	NC 90% (7/8)	NR	[74]
2018, Chen et al.	Urine of healthy and BC patients	80.6%	UETCs	BC 95% (9/20)	NC 80% (16/20)	NR	[61]
2018, Liang et al.	Urine of healthy and BC patients	> 90%	UETCs	BC 71.4% (25/35)	NC 90% (2/20)	NR	[62]
2019, Khoo et al.	BCa cell urine samples spiked	93.30%	EBCC	NR	NR	NR	[134]
2020, Carvalho et al.	BCa cell urine samples spiked	53.3%	UETCs	NR	NR	NR	[65]
2020, Wang et al.	T24 incorporated into standard human blood samples	90%	CTCs	NR	NR	NR	[77]

Abbreviations: BC, BCa; CE, CE (%) = (captured s cells)/ (total input cells) × 100%; CTCs, Circulating bladder tumor cells; EBCCs, Exfoliated BCa cells; EVs, Extracellular vesicles; NC, Normal cells; NR, Not reported; UETCs, Urinary exfoliated tumor cells.

**TABLE 8:** Comparison of microfluidic research with commercial and traditional methods in prostate tumors.

Product or research	Patients or samples, *n*	Capture rate	Targets	Sensitivity	Cost/dollar	Performance	Ref.
Roche assay	10 μL PSA blood samples (*N* = 100).	NR	PSA	NR	NR	The median PSA value of the Roche assay was 1.3 ng/mL with a range of 0.1–67 ng/mL.	[89]
Abbott assay	10 μL PSA blood samples (*N* = 100).).	NR	PSA	NR	NR	The median PSA value of the Abbott assay was 1.2, with a range of 0.3–52.6 ng/mL.	[89]
AdnaTest	Blood samples of PC patients (*N* = 10)	1.03% (primary cancer), 2.92% (metastatic cancer)^a^	CTCs	9/10 (90%)	NR	0.83/mL (primary cancer), 2.29/mL (metastatic cancer)	[110]
Claros	10 μL PSA blood samples (*N* = 100).	NR	PSA	NR	NR	The median PSA is 1.0 ng/mL with a range of 0.2–16 ng/mL	[89]
2017, Renier et al.	Serum samples from PC patients (*N* = 20) and healthy individuals (*N* = 10)	37%/1D, 59%/2D	CTCs	17/20 (85%)	NR	NR	[108]
2019, Obayashi et al.	Metastatic PCa patients (*N* = 14)	PC3:94.60% (PBS); 83.82% (PBS); LNCaP: 82.73% (PBS); 75.78% (blood)^b^	PC3; LNCaP	14/14 (100%)	3000/initial cost, 100/operating cost	NR	[135]
2020, Wang et al.	Serum samples from PC patients (*N* = 10) and healthy individuals (*N* = 8)	73.9%	Exosomes	NR	NR	NR	[102]
2020, Cho et al.	Blood samples of PCa patients (*N* = 14)	2.89% (primary cancer), 4.92% (metastatic cancer)^a^	CTCs	14/14 (100%)	NR	14.8/mL (primary cancer), 20.54/mL (metastatic cancer)	[110]

Abbreviations: CTCs, Circulating bladder tumor cells; EBCCs, Exfoliated BCa cells; EVs, Extracellular vesicles; NC, Normal cells; NR, Not reported; PC, PCa.

^a^ (The number of isolated cytokeratin−positive cells [CTCs])/(Total number of cytokeratin−positive cells [CTCs] and CD45−positive cells [WBCs]) × 100%2.

^b^ The capture efficiency was calculated by counting the number of cells remaining on the chip after sample passage compared with the number of cells that passed through the chip inlet.

In the study of PCa, microfluidic technology is utilized primarily to evaluate biomarkers of PCa cells, including the quantification of PSA, the detection of exosomes, and the separation and enrichment of prostate CTCs. PSA is recognized as an effective tumor marker: a high sample consumption, a single analysis, and a high price limit traditional PSA detection. Microfluidic technology provides a versatile platform for laboratory research that can be optimized for POC equipment portability. In order to respond to high‐level PCa research, scenarios or flexible combinations of analysis have been increased to quantify various PSAs. A second study on the staging of PCa cells demonstrated that microfluidics can be combined with high‐speed cameras to stage PCa cells in various stages of epithelial tumors. In this instance, morphological rheology analysis is utilized. The molecular content of exosomes in the prostate can reveal the specific physiological environment and function of its original cells, such as EpCAM in PCa and non‐cancer exosomes. EpCAM in exosomes of PCa cells and non‐PCa cells differ significantly. Using microfluidic technology in conjunction with magnetic nanoparticles, a microfluidic hybrid platform for Raman detection of tumor cells was developed in this study. It assures the precision and integrity of detection without destroying the body of the chip, demonstrating the potential of microfluidic chips when combined with other technologies. CTCs have emerged as a popular biomarker that could provide genetic and phenotypic characteristics representative for primary and metastatic tumor sites. It has been compared to the FDA‐approved CellSearch System for separating CTCs by microfluidic chip, but it also has some shortcomings. The current research focuses on optimizing the chip's cost, time, and detection efficiency, producing fruitful outcomes. Other studies on CTCs using microfluidic devices and RT‐qPCR have also served as a source of inspiration.

Microfluidic chips for RCa differ from those for BCa and PCa, as it focuses on OOC technology to create an in vitro model to simulate the RCa tumor microenvironment instead of enrichment and evaluation of specific biomarkers. Cell‐free DNA (cfDNA) has aided in the diagnosis, prognosis, and progression monitoring of RCa, as studies have linked the length of cfDNA fragments to carcinogenesis. Researchers have constructed various forms of OOCs or organoid microspheres for anti‐angiogenesis therapy and drug screening using microfluidic technology, as well as created a multi‐organ kidney device to reproduce the spread of kidney cancer to the liver and the role of toxins between intestinal cells and kidney cells. These studies demonstrate the requirement of RCa chips and renal OOCs in developing multi‐organ chips.

### 6.2 Future Perspectives

#### 6.2.1 Generalization

Current microfluidic devices share a similar layout, and their dimensions are designed within a narrow range. Nevertheless, each device could retain specific characteristics in the interface of each channel or the ensuing inspection area, as the chip is typically used as the initial carrier of the detected target, and the entire analysis procedure is typically comprised of multiple processes. In order to match the most common characterization apparatus in the later stage of non‐invasive biopsies, some general standards should be established in the subsequent detection, such as matching standard Ruhr joints and optical detection etc. Multi‐organ chips would lead the future of OOCs. Although the asynchronous growth of different organ cultures in the chip might be a concern, the connecting devices of the flow channel or the conserved transfer valves could be utilized to control the nutrient ratio to achieve synchronized growth of multiple organs in the chip.

#### 6.2.2 Reproducibility

Whether it is a microfluidic chip or an OOC, future microfluidic device development would focus on the data integrity of the chip, and the relevant standards of the general description should also be established to guarantee a concordant data processing procedure. In addition, the manufacturing error of the same chip should be reduced, and the experimental results of the same chip should not vary significantly. The regulation for chip fabrication, surface treatment, and other processes, and future chip fabrication technology must meet the need of mass production requirements. In addition, manipulating high‐throughput organs on a chip with multiple technologies will improve the reproducibility of research data. Furthermore, real‐time monitoring can also guide the researchers in experimenting at the certain time points, which also helps to produce high reproducibility and reduce randomization.

#### 6.2.3 Intelligent Integration

Future research on urinary tumors should attempt to combine microfluidic chip and OOC technology with additional technologies, such as multiple sensors or portable smartphones, which are necessary for POC devices and can perform real‐time, rapid, and multi‐index analysis in different environments at any specific time point. The urinary microfluidic chip is expected to join the current popular artificial intelligence (AI) image algorithm system, which can further enhance the recognition of cell images in the microfluidic channel by automated processing instead of manual counting.

#### 6.2.4 Improving Bionics

One of the most significant issues with urinary tumor OOCs is that the bionic nature of the organs is insufficient, which is common among OOCs for other organ systems. In addition, reports on PCa organ cells are limited. The cell types and organ functions of BCa and kidney cancer organ implants are highly distinct from those of actual human organs. Therefore, the future urinary tumor OOC must first enhance the versatility and bionics of a single organ to accomplish a comprehensive simulation of the function of human organ microenvironment, such as the co‐culture of multiple cell types. For metastatic cancers, it is necessary to study the combination of multi‐organ tumor implants to study a more realistic tumor environment, such as by constructing a cancer model of multiple organs such as the bladder, prostate, renal, and so on.

## 7 Conclusion

In the past decade, significant advances in microfluidic technology have been made in tissue engineering and clinical applications, in addition to the research on the urinary system. Advances in the OOC cover a wide spectrum, including diagnosis and treatment of BCa, screening of PCa, and research on the mechanism of RCa. Due to the limitation of context, the current review may not cover all aspects of the urinary organ, and we hope it could serve as an introductory review for future in‐depth research of OOC toward more practical applications to replace animal experiments, with reduced costs, turn‐around time, and improved effectiveness. We look forward to connecting the entire urinary system organs by combining multiple OOCs to achieve an in vitro model that is more similar to the human urinary system, providing precision and convenience for cancer diagnosis, prognosis and treatment.

## Author Contributions

Jiafu Liu, Xiao Zhi, and Xiaolan Fang were mainly responsible for literature collection, analysis, and manuscript drafting. Wenyao Li, Kaile Zhang, and Wenguo Cui provided critical guidance on the structure and content of the manuscript, as well as thorough revisions. Weixin Zhao, Meng Liu, Enping Lai, and Wenzhuo Fang assisted in literature screening, graphical illustration, and formatting. Yu Zheng and Juan Wang contributed to reference management and proofreading. Jiang Zou and Qiang Fu supported content refinement and helped review the final version.

## Ethics Statement

This review did not involve original animal experiments or human clinical studies. All referenced articles complied with ethical standards for animal welfare and human subject research, including approvals from institutional review boards (IRBs) or equivalent ethics committees, as explicitly stated in their original publications.

## Conflicts of Interest

The authors declare no conflicts of interest.
